# Daily Consumption of Caper Fruit Along With Atorvastatin Has Synergistic Effects in Hyperlipidemic Patients: Randomized Clinical Trial

**DOI:** 10.31661/gmj.v0i0.1345

**Published:** 2019-05-27

**Authors:** Saeed Sardari, Faramarz Fallahi, Fatemeh Emadi, Ali Davati, Narjes Khavasi, Mohammad Gholamifesharaki, Seied Saeid Esmaeili

**Affiliations:** ^1^Department of Iranian Traditional Medicine, Faculty of Medicine, Shahed University, Tehran, Iran; ^2^Department of Persian Medicine, School of Medicine, Zanjan University of Medical Sciences, Zanjan, Iran; ^3^Department of Cardiology Mostafa Khomeini Hospital, Faculty of Medicine, Shahed University, Tehran, Iran; ^4^Department of Biostatistics, Faculty of Medical sciences, Tarbiat Modares University, Tehran, Iran

**Keywords:** Caper, Atorvastatin, Lipid Profile

## Abstract

**Background:**

Dyslipidemia leads to micro- and macro-vascular complications. Atorvastatin is the main therapeutic drug used for dyslipidemia, but it causes side effects such as new type 2 diabetes mellitus onset and elevation of liver enzymes. Herbs may be useful in reducing atorvastatin doses. Caper fruit, an herbal drug in Persian Medicine, has hypolipidemic effects. Hence, the effect of atorvastatin therapy with and without daily caper fruit pickle (CFP) consumption was assessed on hyperlipidemia.

**Materials and Methods:**

In this single-blinded, randomized, controlled trial, 60 hyperlipidemic patients were allocated in two groups and treated with 10 mg atorvastatin plus 40-50 g CFP (A10+CFP) or atorvastatin alone (A10) for eight weeks. Biochemical parameters were measured at baseline, 4, and eight weeks of the intervention. One-way repeated measure ANOVA and mixed ANOVA were used to measure the effect of the two treatments and the interaction between the type of treatment and time on lipid profile.

**Results:**

Serum total cholesterol and low-density lipoprotein cholesterol (LDL-C) were significantly decreased in the A10+CFP group compared with the A10 group (P<0.001 and P<0.001, respectively) from baseline up to the week 8. At week 4, mean changes of LDL-C was significantly higher in the A10+CFP compared with the A10 (P=0.01). Adjusting for the baseline variables, the mean difference of alanine aminotransferase (P<0.01) and triglyceride (P=0.003) were significantly higher in the A10+CFP group at the end.

**Conclusion:**

This study reports that the intake of CFP along with atorvastatin daily may have synergistic effects which improve the lipid profile in hyperlipidemic patients.

## Introduction

Dyslipidemia is the primary enhancer for atherosclerotic cardiovascular diseases (ASCVDs) and occurs before other important risk factors. ASCVDs, a leading cause of mortality worldwide, are inflammatory diseases demonstrating nearly all the characteristics of immune responses, with genetic and environmental factors playing major roles in its etiology. Therefore, the control of dyslipidemia through pharmacotherapy is the primary focus in ASCVD prevention [1]. The most common drugs in the treatment of ASCVDs are statins, especially atorvastatin, which affect the lipid metabolism at the molecular level by inhibiting the activity of 3-hydroxy-3-methylglutaryl-CoA (HMG-CoA) reductase enzyme [2]. However, there is some evidence regarding the effect of atorvastatin on type 2 diabetes mellitus onset and elevation of liver enzymes [3, 4]. The use of herbal medicines may aid in reducing drug doses, subsequently decreasing the side effects of statins. Persian Medicine offers a variety of herbs to control dyslipidemia. Caper (*Capparisspinosa* L.), belonging to the Capparidaceae family, is widely recommended by ITM for treatment of dyslipidemia due to its effect on spleen performance, toxins excretion, and “*Soda*” (*Balgham*) clearance from the liver [5]. Spleen and liver are considered the main organs that control dyslipidemia. One study has demonstrated major roles of caper, including its hepatoprotective, hypolipidemic, anti-diabetic, antioxidant, and anti-inflammatory effects [6]. The blood glucose–lowering effect of caper is due to a reduction in carbohydrate absorption in the intestine, increase in glucose uptake in tissues, glucose depletion from the liver, and regeneration of beta cells of the pancreas [7, 8]. There are some studies on the effects of dietary supplements on lipid profile, as well as liver enzymes [9, 10], but studies on the effects of herbal traditional medicine products along with drugs are rare. In summary, management of blood cholesterol levels is the main focus in primary as well as secondary prevention of atherosclerotic cardiovascular events, such as myocardial infarction and strokes. Both conventional and traditional medicine offer either pharmacological treatment or a vast range of plants and herbal extracts with promising cholesterol-lowering effects. To our knowledge, there is no study on daily caper fruit pickle (CFP) consumption with atorvastatin compared with atorvastatin alone. We aimed to compare the effects of atorvastatin therapy with and without CFP on lipid profile, including total cholesterol, low-density lipoprotein (LDL-C) and high-density lipoprotein cholesterols (HDL-C), and triglyceride (TG) as well as liver enzymes in patients with hyperlipidemia after 8 weeks of intervention.

## Materials and Methods

**Figure 1:**
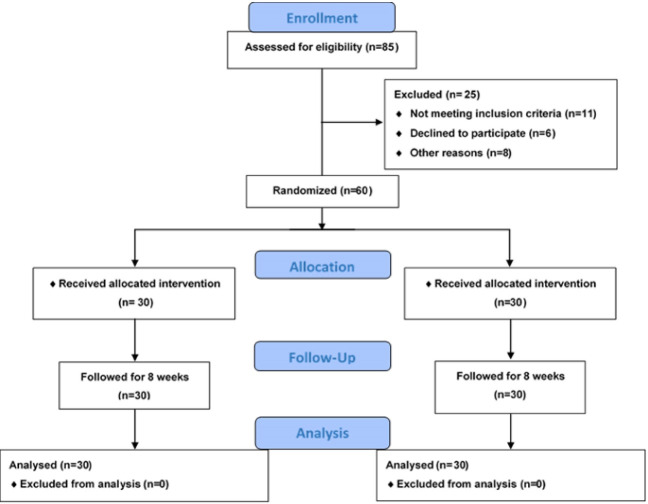
CONSORT flow diagram

A single-blinded, randomized, controlled trial was designed, and the protocol was approved by the institutional review Board of Shahed University (approval code: IR.Shahed.REC.1395.203). Also, the present study registered at the Iranian Registry of Clinical Trials; www.irtc.ir under the code number of IRCT2017032827532N1. CFP preparation and its herbarium code are mentioned in our previous study [11]. Atorvastatin was obtained from Sobhan Daru Company (Rasht Industrial City, Rasht, Iran). The manufacturer performed quality control of the trial medication to guarantee its quality.The participants recruited were men aged 40-60 years who referred to Vali-e Asr Hospital (Zanjan, Iran), were newly diagnosed with hyperlipidemia and were prescribed low-dose atorvastatin (10 mg/day). The recruitment period lasted from March 2017 to January 2018. Hyperlipidemia was defined according to the American Association of Clinical Endocrinologists guidelines [12]. Demographic and clinical data related to hyperlipidemia, including nutritional habits, drug consumption, age, and weight, were obtained by an interviewer according to a checklist. Patients with a positive history of inflammatory diseases, thyroid disorders, and elevated levels of TG (more than 400 mg/L), as well as those who had followed either a vegetarian or a weight-loss diet less than two months before the beginning of the intervention period, were excluded. All patients gave written consent for participation in the study. A total of 60 participants were randomly allocated based on the use of atorvastatin alone (n=30) or CFP along with atorvastatin (n=30) for eight weeks; Figure-1. A specialized nutritionist educated all participants about lifestyle changes by training with the same package. The package included training about a decrease in dietary fat, fast foods, and total calorie intake as well as an increase in dietary fiber. Patients were educated to be physically active 3 times per week for 50 minutes. Then, daily energy intake (from diet) and expenditure (from physical activity) were simulated in all patients. All participants were given 10 mg atorvastatin daily in a single dose, and the side effects of atorvastatin were explained to them. Furthermore, patients allocated to the CFP group were recommended to receive 40-50 g of CFP with one meal on a daily basis. The patients’ adherence to the diet and medication was assessed by telephone interviews once a week. Fasting blood samples were gathered to measure TG, total cholesterol (total-C), HDL-C, LDL-C, alanine aminotransferase (ALT), and aspartate aminotransferase (AST) at the beginning of the study, following 4, and 8 weeks of treatment. The primary outcome was the changes in serum LDL-C levels. Other lipid profiles and liver enzyme tests were considered as secondary outcomes. Testing was conducted by an automated chemistry analyzer using commercially available kits obtained from Pars Azmoon Company (Pars Azmoon Co., Tehran, Iran). By considering the power of 80%, α=0.05, as well as a mean difference of 15.3 mg/dL for LDL-C in the similar previous study [13], 30 patients were needed in each group. Anticipating 15% of dropout, 34 patients were gathered in each group. The Kolmogorov-Smirnov (K-S) test was performed to assess normality, and the Greenhouse-Geisser test was performed to estimate sphericity. Paired-sample *t*-test was applied to determine the alterations from the baseline to the end in each treatment groups, whereas independent sample *t*-test was utilized to evaluate differences between the two groups. Also, one-way repeated measure ANOVA was used to measure the effect of the two treatments, whereas a mixed ANOVA was applied to determine whether any variation in measured parameters is the consequence of the interaction between the type of treatment and time. Therefore, the dependent variables were LDL-C, HDL-C, total-C, TG, ALT, and AST, whereas the within-subject factor was time and the between-subject factor was treatment. All statistical analyses were performed by SPSS 16 (IBM, Armonk, NY, USA), and the statistical significance was considered at less than 0.05.

## Results

Compliance of patients was 90%. As shown in Table-1, the baseline variables did not significantly differ between the two groups (P>0.05). Energy input and physical activity level did not differ between the two groups at the baseline, 4 and eight weeks, significantly (P>0.05). The mean age and weight of participants were 48.6 ± 5.3 years and 87.7 ± 8.2 kg, respectively. For serum levels of LDL-C, total-C, HDL-C, TG, and ALT, the interaction between the type of treatment and time was significant (P<0.001, P=0.001, P<0.001, and P=0.04, respectively). Compared with A10, the A10+CFP group experienced more significant improvement in serum levels of LDL-C (P<0.001), total-C (P<0.001), HDL-C (P=0.008), and TG (P<0.001) at the end of the study. Serum LDL-C level significantly differed between the A10 and A10+CFP groups on weeks 4 and 8 (P=0.01 and P<0.001, respectively). In the A10+CFP group, serum LDL-C and total-C were significantly reduced after four weeks by an average of 26.2 mg/dL and 29.1 mg/dL (P<0.001 and P<0.001), respectively. Additionally, 3.9 mg/dL and 6.1 mg/dL decrease in serum LDL-C and total-C, respectively were observed at week 8 (P<0.001 and P<0.001, respectively). Moreover, serum TG level was significantly reduced by an average of 21.6 mg/dL after four weeks (P=0.003), whereas the difference between weeks 4 and 8 was not statistically significant. In the A10+CFP group, serum ALT level was significantly reduced both at weeks 4 and 8 of treatment (P=0.004 and P=0.01, respectively). The difference in mean of serum AST level was not statistically significant (Table-2).

## Discussion

Atorvastatin therapy along with daily consumption of CFP reduced serum total-C and LDL-C more than the atorvastatin alone in four and eight weeks. Reduction in serum ALT and TG levels was shown at the end of the study and adjusted for the baseline variables. Atorvastatin therapy along with caper had beneficial effects with respect to the lipid profile. One of the side effects in atorvastatin therapy is ALT elevation, which reduced with CFP use after eight weeks. To our knowledge, there is no human trial comparing the effects of CFP consumption with atorvastatin on lipid profile to date. Our results are in agreement with one animal study in which using the aqueous extract of *C. spinosa* reduced lipid profile in both normal and rats with diabetes [7]. Another human study on nonalcoholic fatty liver disease (NAFLD) patients showed the hypolipidemic effect of CFP for eight weeks [11]. The mechanism underlying the lipid-lowering effect of caper fruit is still unknown, but a possible mechanism is that caper causes a reduction in cholesterol absorption from the intestine [14]. It is now well established that LDL-C is a proinflammatory molecule that can directly trigger vascular inflammation. It is demonstrated that oxidized LDL can activate toll-like receptors on macrophages, which triggers inflammatory signaling pathways [15]. Previous studies have reported the anti-inflammatory effect of caper [16-18]. From the ITM perspective, dyslipidemia results from the dysfunction of the stomach and liver, which leads to impaired humor production. It is supposed that caper positively affects *Balgham* levels as one of the four senses of humor, leading to dyslipidemia alleviation [12]. On the other hand, the spleen plays an important role in liver health and humor production [19]. The relationship between splenectomy and hyperlipidemia has been reported [20]. In a review study by Tarantino *et al*., the importance of the spleen in NAFLD is examined. According to their findings, spleen dysfunction is very important in metabolic disorders and obesity [21]. Spleen size has an inverse association with lysosomal lipase activity, which leads to dyslipidemia [22].Moreover, several studies indicate that statin therapy causes various side effects such as neurological symptoms, liver damage, and myalgia [23, 24]. It is also demonstrated that the use of statin is associated with a moderate increase in the risk of developing new-onset type 2 diabetes [3, 4]. Results of previous studies are inconsistent regarding whether these side effects are dose-related or not. Evidence-based data showed that statins increase the risk of diabetes mellitus onset at higher doses [25], whereas others did not show this effect [26-28]. According to our results, an increase in the efficacy of the least dose of statin therapy through the use of caper can motivate the avoidance of higher doses of the drug. This is the first randomized controlled trial that assessed the combined effects of herbal medicine and atorvastatin on hyperlipidemia. Despite the widespread application of statins, their lipid-lowering effects vary widely among individuals. As a medicinal herb, caper can provide an alternative remedy for dyslipidemia. The main advantages of CFP are its wide availability, affordability, effectiveness, and safety. There are some limitations. First, all patients consumed nearly the same quantity of CFP, which was not adjusted to their weight. Therefore, the amount of the CFP used (in grams) per 1 kg weight may differ across patients, negatively affecting our results. Also, we only enrolled male participants in this study, and thus, we are unable to claim that CFP exerts the same effects on females. Moreover, another study with a group receiving CFP alone can determine the independent consequence of CFP on lipid management.

## Conclusion

This study proposes that a combination of conventional (atorvastatin) and traditional medicine (CFP as the herbal drug) to treat dyslipidemia has more beneficial effects compared with atorvastatin alone. Identification of the molecular mechanisms underlying the lipid-lowering effects of caper can open new windows in the application of herbal medicine to treat dyslipidemia.

## Acknowledgment

Authors are very thankful from all patients participated in the present study. This study was supported financially under grant: No. IR.Shahed.REC.1395.203.

## Conflict of Interest

We have no conflict about this study.

**Table 1:** Baseline Characteristics of Patients. All Data Are Expressed as Mean± SD

**Variables** ^a^	**A10(N=30)**	**A10+CFP(N=30)**
**Age(year)**	49.4±4.9	47.8±5.6
**Weight(Kg)**	87.9±8.3	87.6±8.2
**LDL-C(mg/dl)**	152.5±22.4	153.2±23.1
**Total-C(mg/dl)**	232.6±24.5	238.5±28.1
**HDL-C(mg/dl)**	36.7±8.5	39.1±13.1
**TG(mg/dl)**	218.8±68.5	227.8±103.3
**ALT(U/L)**	33.2±10.7	31.7±10.1
**AST(U/L)**	26.5±6.9	23.2±6.6

**Total-c:** total cholesterol; **TG:** triglyceride; **ALT:** alanine aminotransferase; **AST:** aspartate aminotransferase a P>0.05 assessed by independent sample t-test

**Table 2:** Changes in Lipid Profile within and Between the Studied Groups. All Data Are Expressed as Mean± SD

**Groups**		**Total-C** **(mg/dl)**	**LDL-C** **(mg/dl)**	**HDL-C** **(mg/dl)**	**TG** **(mg/dl)**	**ALT** **(U/L)**	**AST** **(U/L)**
**A10**	**Baseline vs. 4** ^th^ ** week**	-29.1±2.7^a^	-26.2±2.4^a^	1±0.4	-21.6±4.7^a^	-0.07±0.9	-0.1±0.6
	**4** ^th^ ** week vs. 8** ^th^ ** week**	-6.1±1.3^a^	-3.9±1^a^	0.6±0.5	-5.8±2.9	-2.4±0.6^b^	-0.5±0.5
	**Baseline vs. 8** ^th^ ** week**	-35.2±3.1^a^	-30±2.5^a^	0.4±0.7	-27.4±6.6^a^	-2.3±1.3	-0.4±0.8
**A10+CFP**	**Baseline vs. 4** ^th^ ** week**	-48.9±4.4^a^	-38.4±3.7^a^	-0.2±1.9	-48.1±13.1^b^	-4.64±1.3^b^	-0.49±0.77
	**4** ^th^ ** week vs. 8** ^th^ ** week**	-12.5±2.1^a^	-12.1±1.5^a^	-0.8±0.3	-7.6±3.7	-1.57±0.87	-0.43±0.64
	**Baseline vs. 8** ^th^ ** week**	-61.4±5.1^a^	-50.6±4.1^a^	0.6±1.9	-55.7±15.4^b^	-6.21±1.94^b^	-0.93±1.14

**Total-C:** total cholesterol; **TG:** triglyceride; **ALT:** alanine aminotransferase; **AST:** aspartate aminotransferase a P≤0.001; b P≤0.01
